# Multiple Incidental Unruptured Splenic Artery Aneurysms Following Severe Trauma

**DOI:** 10.7759/cureus.11136

**Published:** 2020-10-24

**Authors:** Mohamed Selim, Amr Awad Albayomy, Leena A Almuhaish, Shouq A Alraddadi, Wasan M Alharbi

**Affiliations:** 1 Vascular Surgery, King Fahad University Hospital, Dammam, SAU

**Keywords:** splenic artery aneurysm, unruptured aneurysm, splenic artery, incidental aneurysm

## Abstract

Splenic artery aneurysm is a relatively rare, potentially life-threatening condition due to its high potential for rupture. We report a case of two incidentally found unruptured splenic artery aneurysms in a 39 year-old female victim of severe trauma resulting from a motor vehicle accident (MVA). Management by coiling of the splenic artery aneurysm and embolization to the entire segment distal to it was successfully performed for this patient.

## Introduction

The splenic artery is the third most common artery affected by an aneurysm following aorta and iliac arteries and it accounts for nearly all cases of visceral artery aneurysms. True splenic artery aneurysm (SAA) is a rare pathology that carries a high risk of morbidity and mortality if it ruptures. The prevalence of SAA is reported to be less than 1%; furthermore, it is found to be four times more in females than males. Most cases of SAA go unrecognized due to its asymptomatic course and it is usually an incidental finding on imaging modalities [1-3].

The etiology of SAA remains unclear nevertheless, multiple risk factors take a part in the pathogenesis of SAA such as pregnancy, portal hypertension, atherosclerosis, pancreatitis, and abdominal trauma. Due to the increasing usage of CT in medical practice, the detection of asymptomatic aneurysm cases is rising. In case of a symptomatic aneurysm, the presenting symptoms mainly include vague epigastric pain or left upper quadrant pain radiating to the left shoulder; due to the rare nature of SAA, it is not usually suspected in the first presentation [2,3].

Ultrasonography is used as an initial evaluation tool while CT with contrast is used for identification. However, digital subtraction angiography is the gold standard for diagnosis of SAA. Regarding the management of SAA, no clear guidelines were proposed till now and indications for treatment remain contentious. However, it is recommended to intervene rather than to observe the patient in case of an aneurysm with a size of more than 2cm or in symptomatic patients, pregnant patients, females of childbearing age.The approaches to treat SAA include surgical repair (either open or laparoscopic), endovascular repair, and conservative measures. The choice of treatment method depends on the aneurysm characteristics, the patient’s operative risk, condition and comorbidities [2,3]. 

Here, we report a case of an incidental SAA diagnosed in a 39 years-old female victim of MVA treated successfully with coiling.

## Case presentation

**Figure 1:**
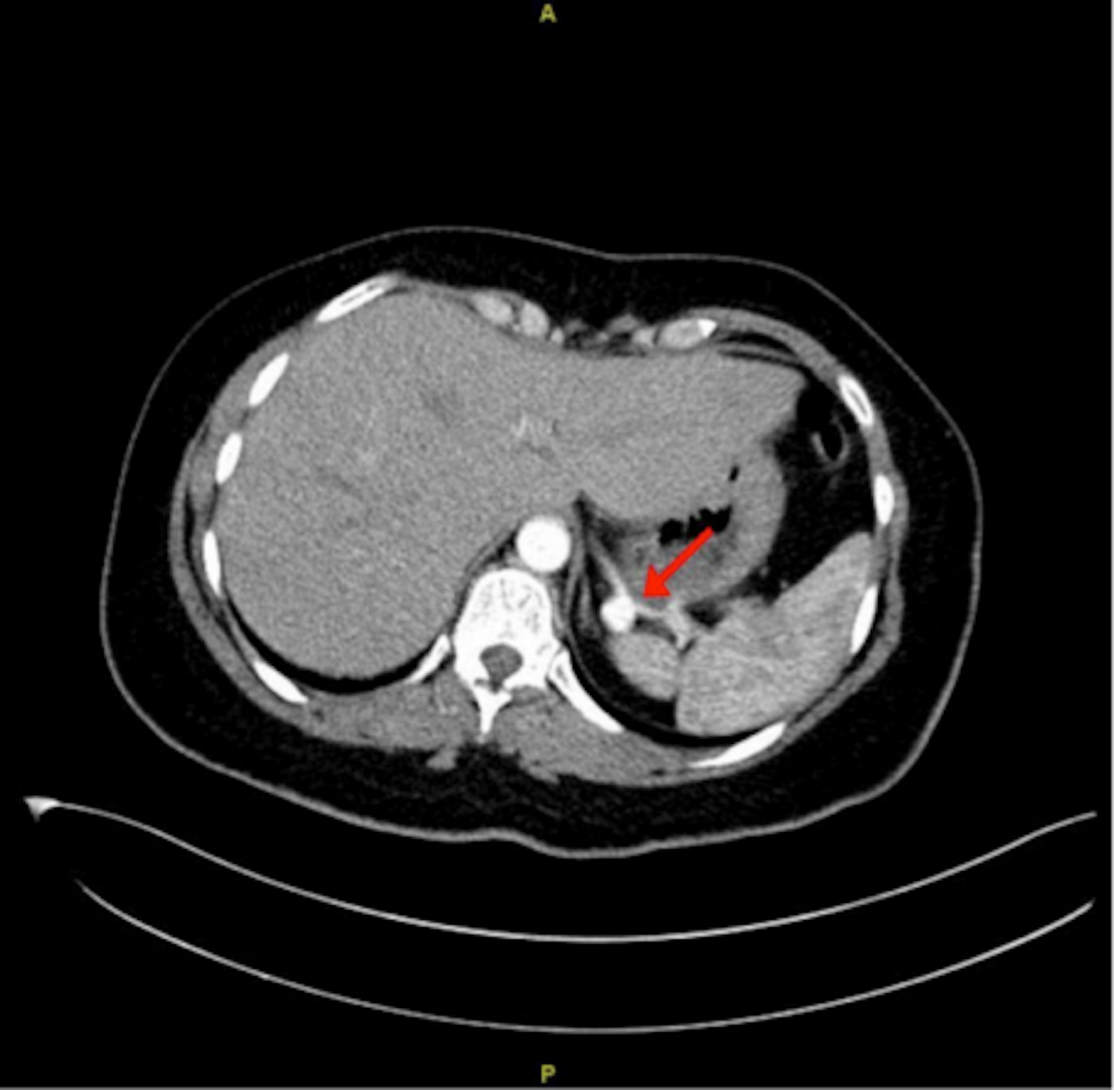
CT scan revealing an unruptured mid to distal splenic artery aneurysm

**Figure 2:**
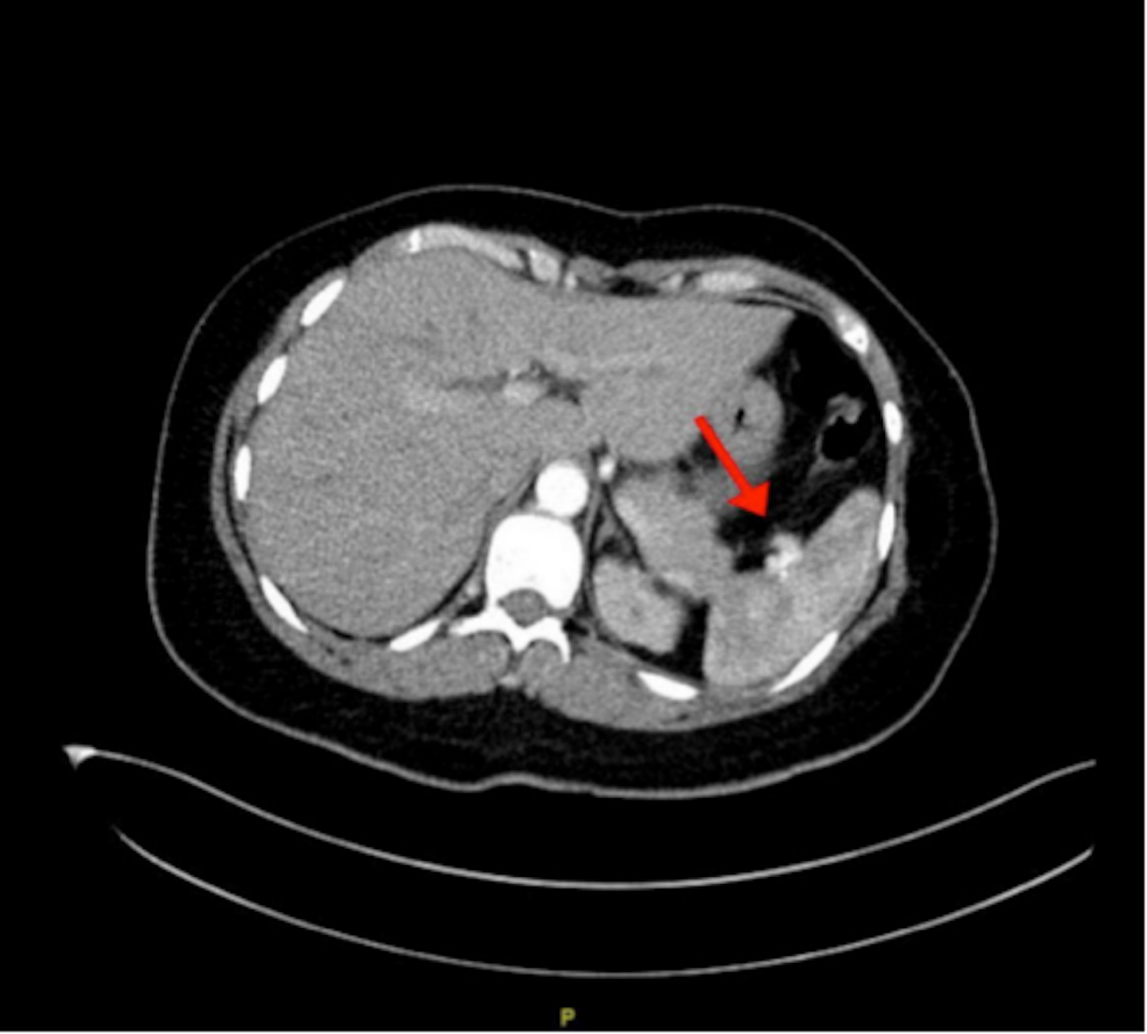
CT scan revealing an unruptured hilar splenic artery aneurysm

A 39-year-old married Saudi female, not previously known to have any medical illnesses, was brought by an ambulance after suffering an MVA. The patient was in the driver’s seat wearing a seat belt when a truck collided with her from the back and rolled her over. The patient was not ejected from the car. She was vitally stable upon presentation. Primary survey and secondary survey were done and hip tenderness was observed. CT pan-scan with contrast was done and it showed two splenic artery unruptured saccular aneurysms, one located at mid to distal splenic artery (Figure 1) and the other at the hilum (Figure 2).

**Figure 3:**
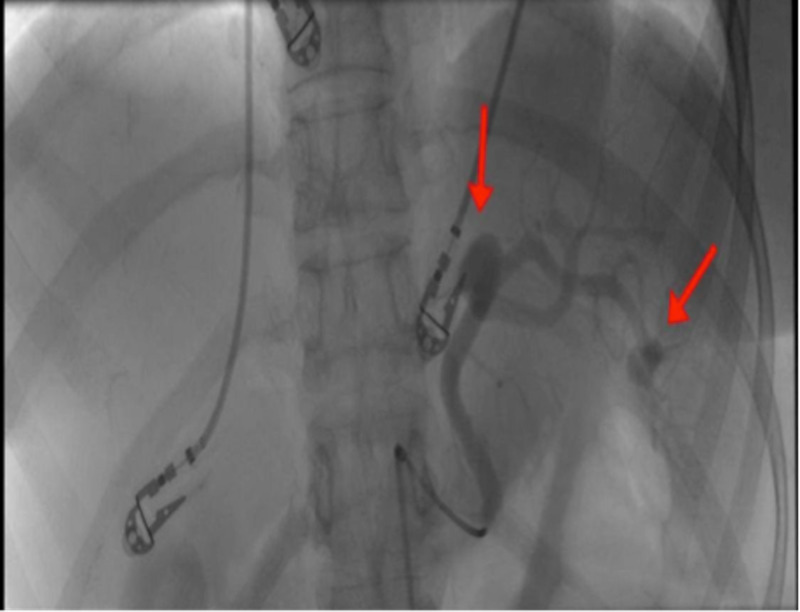
Splenic artery angiogram revealing two splenic artery aneurysms

In addition, it showed a fractured left anterior sixth rib and a fractured left posterior 12th rib as well as a fractured left sacroiliac joint and symphysis pubis. Hemoglobin and hematocrit levels were stable indicating the absence of any active major internal bleeding despite severe trauma. Splenic artery angiogram and embolization were done under conscious sedation with local anesthesia. A 6 French sheath was placed retrograde through the right common femoral artery and a saccular dilatation of 1.5 cm in maximum dimension was seen mid to distal splenic artery along with a less than 1 cm saccular hilar aneurysm (Figure 3).

**Figure 4:**
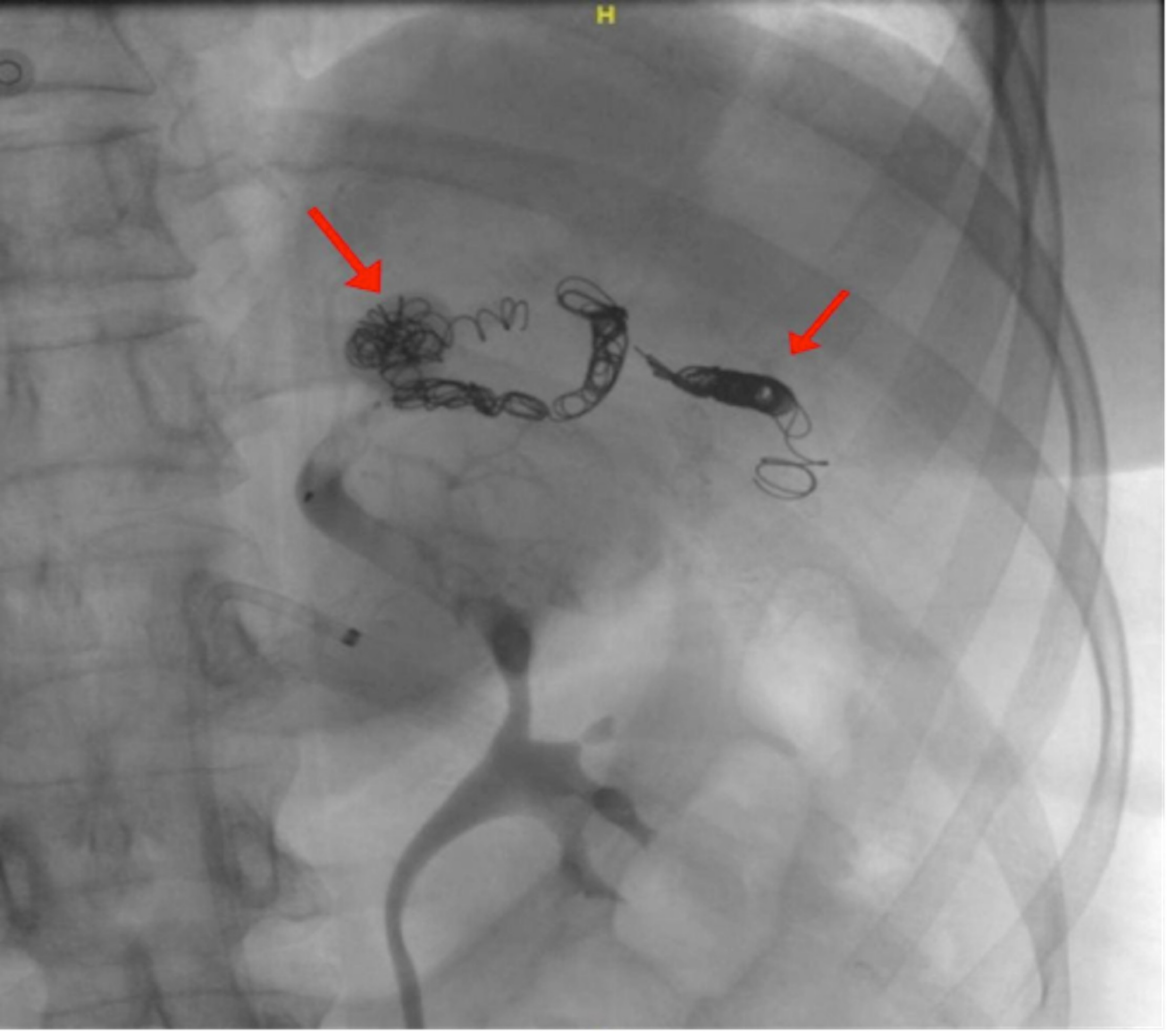
Splenic artery angiogram revealing coiled splenic artery aneurysms

Coiling by closing afferent and efferent vessels was done to the larger aneurysm. As for the distal aneurysm and due to its location in the hilum of the spleen, management was done through embolization to the rest of the splenic artery segment distal to the larger aneurysm utilizing multiple detachable coils and a vascular plug (Figure 4).

A post-management angiogram showed complete isolation of the aneurysm and no blood filling was seen. The right common femoral access sheath was removed and an angio-seal closure device was utilized to maintain homeostasis. No complications arose during or after the procedure and successful embolization was achieved. Post-operatively, the patient was put under observation for 48 hours . A follow-up CT scan was planned to be done 1 month after, as well as follow up with abdominal ultrasonography to rule out any complications of the procedure in the form of splenic necrosis, abscess or peritonitis.

## Discussion

Splenic artery aneurysm, although rare, is the most common type of arterial visceral aneurysm [4], and the second most common abdominal aneurysm [5]. Our patient is female, consistent with the literature, in which this pathology is four times more common in females than males [4]. Most splenic artery aneurysms are asymptomatic prior to rupture. However, patients may present with variable nonspecific gastrointestinal symptoms, the most common ones being vague or sharp epigastric or left upper quadrant pain that may radiate to the left shoulder [2]. Diagnosis is considered difficult due to absence of clinical manifestations; most cases are detected incidentally in patients undergoing abdominal imaging studies for other indications [6]. Cystic lesions found by ultrasound are very suggestive of SAA. However, further investigations are required to rule out other conditions. On the other hand, CTA or MRA are sufficient to diagnose a SAA [7]. The patient in this case report was asymptomatic and the SAA was detected incidentally as she underwent CT pan-scan after she suffered an MVA. In general, intervention is indicated due to high risk of aneurysmal rupture for 2 cm aneurysms, or in symptomatic patients, cirrhotic patients who are liver transplant candidates, or women who are pregnant or of childbearing age as in this patient. Different approaches are used to treat splenic artery aneurysms. The gold standard is an open surgical approach [8,9]. However, endovascular approach is currently preferred as it has a lower risk of morbidity and mortality with faster recovery, and shorter hospital stay [3]. Endovascular techniques include stent placement or coiling techniques. Stent placement is used for fusiform true aneurysms. On the other hand, coiling techniques are used for saccular aneurysms [2]. Major complications arising from coiling techniques such as peritonitis and sepsis are rare but potentially life-threatening [10]. An endovascular approach with coiling was used for this patient and no postoperative complications were observed. Due to the presence of two different SAA in this patient, management was slightly more complicated and an endovascular approach with coiling to the larger aneurysm and embolization to the rest of the splenic artery segment distal to it was done and no postoperative complications were observed. Nevertheless, follow up with CT or Doppler US is required in subsequent months to evaluate therapeutic efficacy [11].

## Conclusions

Splenic artery aneurysm is a very rare condition with high risk of mortality, especially following an aneurysmal rupture primarily among pregnant patients. Therefore, it is essential to obtain a diagnosis. However, it requires a high level of suspicion, as most patients are clinically silent. Splenic artery aneurysm rupture should be considered in any female of child-bearing age presenting with sudden cardiovascular collapse. Endovascular treatment is currently preferred over laparotomy as it has less complications and shorter hospital stay. Unless there is hemorrhagic shock, laparotomy is used. It is still not clear which endovascular technique is the best, most effective, and safest.
